# A bibliometric and visualization analysis for global research trends in Wushu and mental health (1981–2024)

**DOI:** 10.3389/fpsyt.2026.1737574

**Published:** 2026-02-13

**Authors:** Shu Chen Liu, Kenny S. L. Cheah, Syed Kamaruzaman Bin Syed Ali, Hui Min Qu, Zhen Lin Wang

**Affiliations:** 1 Department of Mathematics and Science Education, Faculty of Education, Universiti Malaya, Kuala Lumpur, Malaysia; 2 Department of Education Management, Planning and Policy, Faculty of Education, Universiti Malaya, Kuala Lumpur, Malaysia; 3 Department of Educational Foundations & Humanities, Faculty of Education, Universiti Malaya, Kuala Lumpur, Malaysia; 4 Centre for Sustainable Urban Planning and Real Estate (SUPRE), Faculty of Built Environment, Universiti Malaya, Kuala Lumpur, Malaysia

**Keywords:** bibliometrics, complementary and integrative medicine, global trends, mental health, mind–body interventions, public health, Wushu

## Abstract

**Background:**

Mental health has become one of the most urgent public health issues in the 21st century, and the COVID-19 pandemic has significantly increased this problem. As a traditional mind-body practice, Wushu (e.g., Tai Chi, Qigong) is increasingly recognized for its therapeutic potential in mental health. However, bibliometric studies in this eld remain scarce.

**Methods:**

This study aims to visualize the Wushu and mental health (WMH) related research through bibliometric analysis of the Web of Science database (1981–2024). It examines publication trends, core journals, international collaboration, leading authors, and thematic evolution. A systematic search using Boolean operators identified 536 articles. To conduct a complementary analysis of the findings, this study compared the 23 clinical trials identified from PubMed (2020–2024) with the research trends obtained from the bibliometric analysis.

**Results:**

The study found that the number of published articles and cited times increased significantly in the past five years, which confirmed the influence of COVID-19 in this field. China and the United States, represented by Harvard University, are the main pushing forces in this area. The research focus has shifted from rehabilitation orientation to comprehensive mental and public health perspectives. Future development trends may include strengthening international cooperation, standardizing intervention programs, and cross-cultural research.

**Conclusion:**

This multi-database analysis provides researchers and policymakers with a scientific reference for the WMH field. It clearly reflects current research trends and future research directions in WMH.

## 1 Introduction

Mental health has become one of the most pressing public health challenges of the 21st century (1). According to World Health Organization (WHO) data, approximately one in eight people worldwide (about 970 million individuals) suffered from mental disorders in 2019, with anxiety and depression being the most prevalent (2). The COVID-19 pandemic has further exacerbated this issue, with cases of anxiety and major depression increasing by 26% and 28%, in 2020 alone (2).

With the persistent rise in the incidence of psychological disorders, an increasing number of scholars and clinical researchers are exploring intervention approaches beyond pharmacological treatments (3). Among numerous non-pharmacological therapies, Chinese Wushu including tai chi, qigong, and other martial arts practices is growing in recognition as an effective mind-body intervention for promoting mental health (4, 5). As a mind-body practice integrating physical movement, breath regulation, and meditative focus (6), Wushu demonstrates significant therapeutic potential in depression, anxiety, and quality of life (7, 8). Its accessibility, low cost, and cultural philosophy emphasize its relevance as a complementary strategy for mental health (9).

Over the past several decades, researchers have conducted multidimensional explorations into the psychological benefits of Wushu interventions, encompassing study types such as randomized controlled trials (RCTs) (10), systematic reviews (11), and meta-analyses (12). These studies support the positive effects of Wushu among diverse populations, demonstrating its wide-ranging possibilities for application in emotional regulation, cognitive enhancement, and psychological recovery (3, 5, 7). Therefore, the publications in this field have significantly increased in recent years, reflecting sustained global academic interest in Wushu and Mental Health (WMH).

However, despite extensive research revealing the psychological intervention value of Wushu, the overall global burden of mental health issues continues to rise (1). Numerous RCTs, systematic reviews, and meta-analyses have provided positive experimental results, but this evidence hasn’t been effectively translated into mainstream mental health services or large-scale public intervention practices (13, 14). This study, through systematic literature review, found that the lack of a comprehensive analysis of the current state of research on WMH is probably one of the key reasons for the gap in consensus within this field. Although several reviews have attempted to evaluate intervention effectiveness, little is known about the overall development progress in this field (15). This gap hinders researchers identifying key research priorities and standardizing study designs.

To address this gap, this study employs bibliometric and visualization analysis methods to systematically survey global research findings on WMH. The objective is to reveal the overall research trends and evolution in this field, providing a theoretical foundation and practical direction for the scientific application of Wushu in mental health interventions. Specific objectives include:

1. Charting the publications and citations trend of WMH.
2. Identifying key research nations, institutions, journals, and core authors.
3. Analyzing research hotspots and changes in mental health themes.
4. Summarizing current research limitations and development directions to provide systematic academic references for subsequent studies.

## 2 Methods

**Figure 1:**
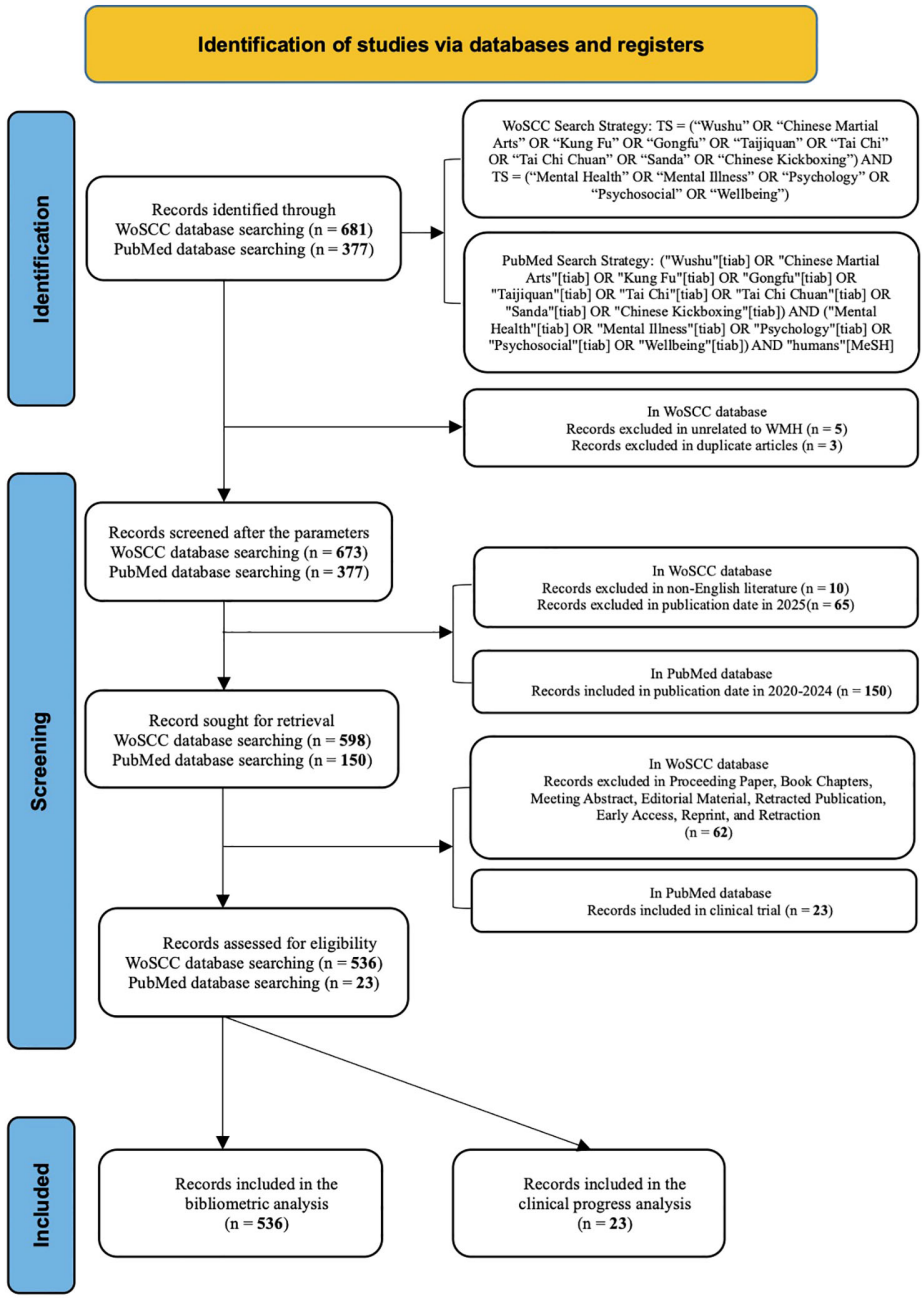
Flow chart of the article selection for WMH research.

As shown in **Figure 1**, this study followed the PRISMA 2020 (16) reporting framework to enhance transparency and reproducibility. Employing a mixed-methods framework from multiple databases, it combined bibliometric analysis with systematic review validation. The bibliometric analysis aimed to provide a knowledge structure and thematic evolution of WMH research (17). However, the systematic review used trial results collected in PubMed to validate clinical progress over the past 5 years (2020–2024).

### 2.1 Bibliometric analysis

Bibliometric analysis is a quantitative academic evaluation method that presents the developmental trends within specific research field (18). Through statistical techniques, this approach measures the scale of research output, identifies emerging trends, maps knowledge landscapes, and key influential authors, institutions, and publications (17–20). Compared to systematic reviews, bibliometric analysis offers a holistic view (19).

The bibliometric analysis aims to provide the knowledge structure and thematic evolution of WMH research. The data for the bibliometric analysis were sourced from the WoSCC, a major multidisciplinary database renowned for its comprehensive coverage of high-quality peer-reviewed journals across science, social sciences, arts, and humanities (20). Its powerful indexing and citation data render it particularly suitable for bibliometric research (20). As this study utilized publicly available data from WoSCC and didn’t involve any human or animal subjects, no ethical approval was required (18, 20).

#### 2.1.1 Data collection and search strategy

A systematic search strategy was devised utilizing Boolean operators to retrieve the relevant literature (20). The search was executed within the WoSCC database in September 2025. To ensure search precision, the selection of subject terms referred to two previous systematic reviews (15, 21). These provided clear keyword boundaries for constructing this search strategy. Therefore, we applied the following Boolean expressions:

TS = (“Wushu” OR “Chinese Martial Arts” OR “Kung Fu” OR “Gongfu” OR “Taijiquan” OR “Tai Chi” OR “Tai Chi Chuan” OR “Sanda” OR “Chinese Kickboxing”) AND TS = (“Mental Health” OR “Mental Illness” OR “Psychology” OR “Psychosocial” OR “Wellbeing”).

The initial search yielded 671 records. First, duplicate records were removed using Zotero software, excluded 3 publications. Subsequently, to construct a complete analytical dataset for the target period (1981–2024), records published in 2025 (n=65) were excluded. To further ensure linguistic consistency, non-English literature (n=10) was also excluded. Among the remaining literature, this study excluded records of the following article types, including proceeding paper (n=19), book chapters (n=6), meeting abstract (n=24), editorial material (n=1), retracted publication (n=6), early access (n=3), reprint (n=2), and retraction (n=1). Subsequently, two researchers independently reviewed abstracts and conclusions, and after discussion, excluded 5 articles unrelated to the WMH theme. Ultimately, 536 publications were included in the bibliometric analysis.

### 2.2 Systematic review of clinical trials

This systematic review aims to summarize clinical progress in the field of WMH over the past five years (2020–2024). Research data were collected from the PubMed database. Eligible studies were required to meet the following criteria: (1) clinical trial design; (2) human participants; (3) interventions involving Wushu related practices; (4) outcomes related to psychological or mental health; (5) articles published in English; and (6) publication between 2020 and 2024. To ensure consistency regardless of the database, we selected the same terms. The specific search strategy is as follows:

(“Wushu”[tiab] OR “Chinese Martial Arts”[tiab] OR “Kung Fu”[tiab] OR “Gongfu”[tiab] OR “Taijiquan”[tiab] OR “Tai Chi”[tiab] OR “Tai Chi Chuan”[tiab] OR “Sanda”[tiab] OR “Chinese Kickboxing”[tiab]) AND (“Mental Health”[tiab] OR “Mental Illness”[tiab] OR “Psychology”[tiab] OR “Psychosocial”[tiab] OR “Wellbeing”[tiab]) AND “humans”[MeSH].

The search findings showed 377 studies focused on the WMH field. Study selection was conducted independently by two reviewers, and disagreements were resolved through discussion until consensus was reached. The included studies were limited to clinical trials published within the past five years (2020–2024). Ultimately, 23 studies were selected for systematic analysis. Given the exploratory scope of this review and the heterogeneity of study designs and outcome measures, a formal risk-of-bias assessment was not conducted.

### 2.3 Data visualization

This study employed bibliometric methods to conduct multidimensional analyses in the WMH field, including publication and citation trends, core journals, country/region contributions, institutional collaboration networks, and thematic evolution. We extracted records (Plain Text File format) for 536 final qualifying publications from the WoSCC and used Microsoft Excel 2019 for descriptive statistics (20).

For geographic visualization, the online tool Datawrapper was employed to generate a global publication distribution map (18). Furthermore, Biblioshiny (the interactive interface of the Bibliometrix R package) was utilized for Bradford’s Law analysis, collaboration network construction, keyword co-occurrence visualization, and thematic evolution trend visualization (18, 20).

## 3 Results

### 3.1 Analysis of publication and citation trends

**Figure 2:**
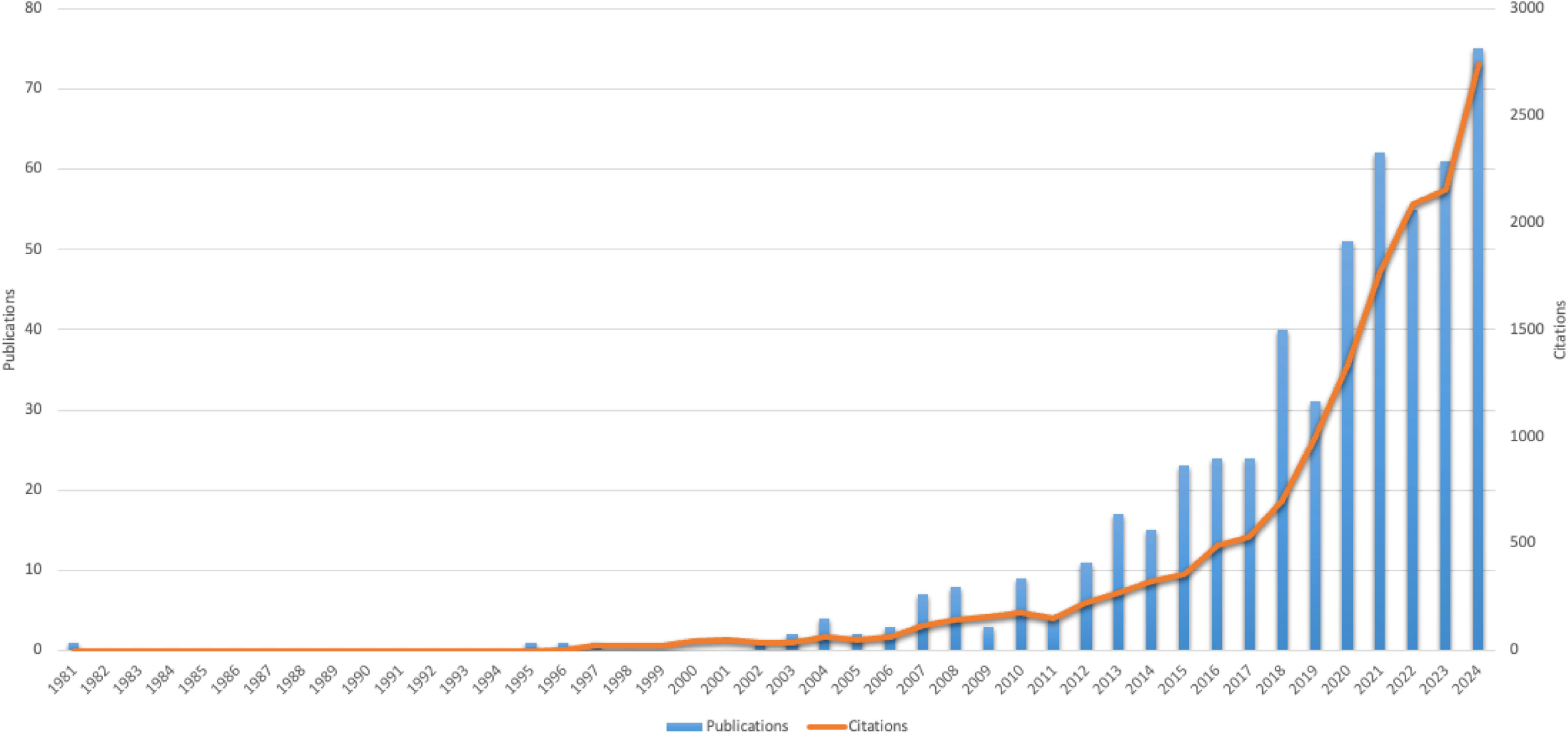
Annual trends of publications and citations in WMH research.

Based on the previously defined search strategy, WoSCC indexed a total of 536 publications related to WMH between 1981 and 2024. **Figure 2** comprehensively illustrates the annual publication volumes and citation counts within the WMH research field during this period. Publications on this topic received combined citations totaling 17,388, achieving an average citation count of 32.44 per article and an H-index of 64. During the initial phase (1981–1999), both publication volume and citation counts remained low, averaging fewer than 1 article and 4 citations annually. After 2000, publication numbers gradually increased. The annual output first reached double digits in 2012 (n=11), after which both publications and citations showed a stable growth. Over the past five years (2020–2024), an average of 60 articles per year have been published on WMH. In 2024, 75 articles were published in this field, representing a threefold increase from a decade ago. Meanwhile, the number of citations reached its peak at 2,739 that year.

### 3.2 Journals and literature sources

**Figure 3:**
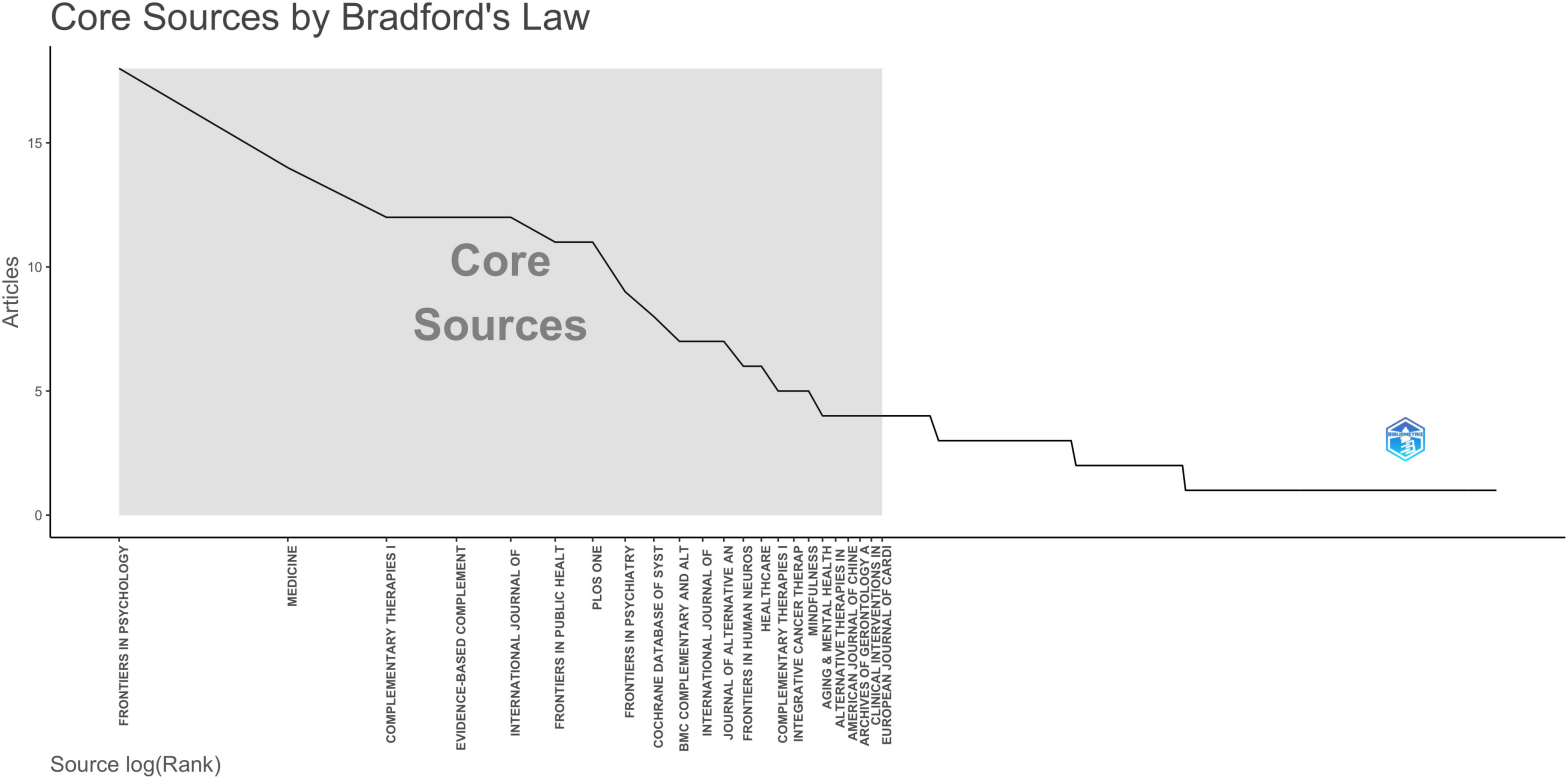
Bradford’s Law presents 23 core journals on WMH.

In bibliometric research, Bradford’s Law is widely applied to identify core journals within specific scientific fields, providing a crucial framework for distinguishing high-output publication sources from marginal ones (18, 20). Based on Bradford’s Law, a total of 23 journals were identified as core sources for WMH research (**Figure 3**). In total, the 287 journals published WMH-related research. Core journals constituted only 8% of the source but contributed 33% of the total publications (n=179).

To further identify the primary channels for WMH academic research, we selected 10 most relevant journals for detailed analysis (**Table 1**). Journal impact indexes are obtained from the Journal Citation Reports (JCR) 2024 edition, including the Journal Impact Factor (JIF) and subject quartiles, to evaluate the academic position of relevant journals. Collectively, these journals contributed 21% of the total research output (n=114). Most journals publishing WMH research were ranked in the Q1 or Q2 categories, reflecting a strong presence in high-quality academic venues. Among them, three journals belonged to the Integrative & Complementary Medicine category, while two were classified under Medicine, General & Internal and Public, Environmental & Occupational Health. Their Impact Factors (IF) ranged from 1.4 to 9.4, with the Cochrane Database of Systematic Reviews holding the highest impact factor.

**Table 1:** Top 10 journals publishing WMH-related research.

Journal	Articles (%)	IF 2024	CQ 2024	Publisher name	JCR category
Frontiers in Psychology	18 (3.358%)	2.9	Q1	Frontiers Media SA	Psychology, Multidisciplinary
Medicine	14 (2.612%)	1.4	Q2	Lippincott Williams & Wilkins	Medicine, General & Internal
Complementary Therapies in Medicine	12 (2.239%)	3.5	Q1	Churchill Livingstone	Integrative & Complementary Medicine
Evidence Based Complementary and Alternative Medicine	12 (2.239%)	N/A	N/A	Hindawi LTD	Integrative & Complementary Medicine
International Journal of Environmental Research and Public Health	12 (2.239%)	N/A	N/A	MDPI	Public, Environmental & Occupational Health
Frontiers in Public Health	11 (2.052%)	3.4	Q1	Frontiers Media SA	Public, Environmental & Occupational Health
Plos One	11 (2.052%)	2.6	Q2	Public Library Science	Multidisciplinary Sciences
Frontiers in Psychiatry	9 (1.679%)	3.2	Q2	Frontiers Media SA	Psychiatry
Cochrane Database of Systematic Reviews	8 (1.493%)	9.4	Q1	Wiley	Medicine, General & Internal
BMC Complementary and Alternative Medicine	7 (1.306%)	4.782	Q1	BMC	Integrative & Complementary Medicine

If, Impact Factors; Cq, Category Quartile; Jcr, Journal Citation Report.

Interestingly, among the top ten most highly cited articles (**Table 2**), four explicitly mention the research type “Systematic Reviews” in their titles. The most frequently cited article, *Reducing Frailty and Falls in Older Adults: An Investigation of Tai Chi and Computerized Balance Training* (22), published in 1996, has been cited 727 times, approximately three times more than the tenth-ranked article. Among these influential works, the journal with the highest impact factor is the Journal of the American College of Cardiology (IF = 22.3, CQ=Q1). Overall, six of the ten (60%) articles were published in JCR Q1 journals, and more than half were authored by researchers from the United States.

**Table 2:** Top 10 most highly cited articles on WMH.

TC	Article title	Journal	Published year	Country	IF 2024	CQ 2024
727	Reducing frailty and falls in older persons: An investigation of Tai Chi and computerized balance training (22)	Journal of the American Geriatrics Society	1996	USA	4.5	Q1
564	Physical activity and exercise for chronic pain in adults: an overview of Cochrane Reviews (23)	Cochrane Database of Systematic Reviews	2017	UK	9.4	Q1
541	Physical Activity Interventions for People with Mental Illness: A Systematic Review and Meta-Analysis (12)	Journal of Clinical Psychiatry	2014	Australia	4.6	Q1
437	Effectiveness of physical activity interventions for improving depression, anxiety and distress: an overview of systematic reviews (24)	British Journal of Sports Medicine	2023	Australia	16.2	Q1
335	The effect of Tai Chi on health outcomes in patients with chronic conditions - A systematic review (25)	Archives of Internal Medicine	2004	USA	N/A	N/A
328	Physical exercise as non-pharmacological treatment of chronic pain: Why and when (26)	Best Practice & Research in Clinical Rheumatology	2015	USA	4.8	Q1
309	A systematic review of physical activity and quality of life and well-being (27)	Translational Behavioral Medicine	2020	USA	3	Q2
305	Positive Psychological Well-Being and Cardiovascular Disease JACC Health Promotion Series (28)	Journal of the American College of Cardiology	2018	USA	22.3	Q1
279	Low back pain in older adults: risk factors, management options and future directions (29)	Scoliosis and Spinal Disorders	2017	China	N/A	N/A
216	Evidence-Based Nonpharmacologic Strategies for Comprehensive Pain Care: The Consortium Pain Task Force White Paper (30)	Explore: The Journal of Science & Healing	2018	USA	2.2	Q2

TC, Total Citation; IF, Impact Factors; CQ, Category Quartile.

### 3.3 Geographic distribution and institutional contributions

**Figure 4:**
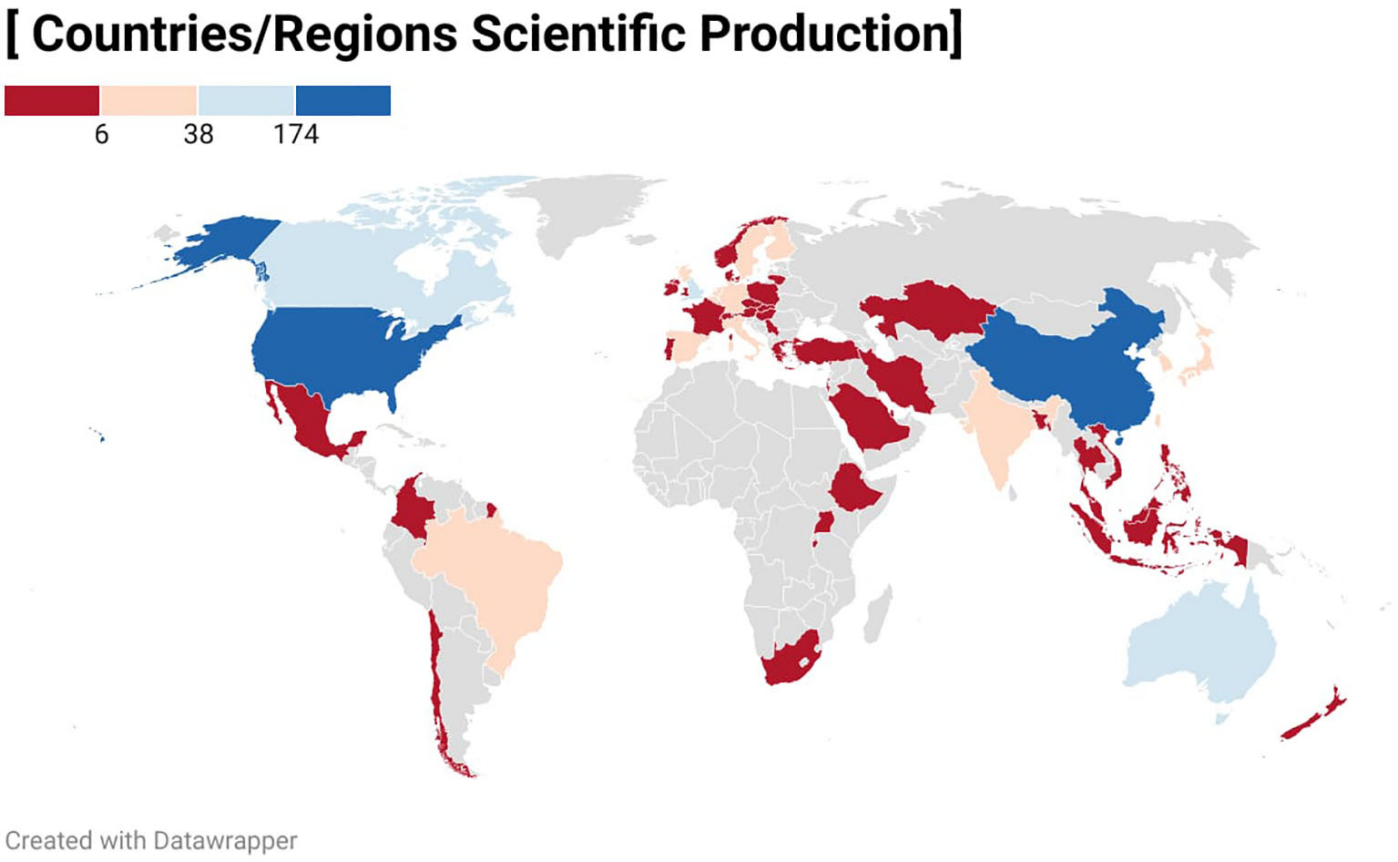
Global distribution of WMH publications by country/region.

**Figure 4** was generated using the Winkel Tripel projection to minimize distortions in area, direction, and distance. Different color bands represent the number of publications from each country. In fact, there are 58 countries/regions worldwide that have made academic contributions to the development of this field. It shows the global distribution of WMH related publications, where the dark blue countries represent China (n=202) and the United States (n=174), each accounting for over 30% of the total publications.

As shown in **Table 3**, among the top ten countries by publication volume, citation counts exhibit significant differences. China produced the highest number of publications (n=202) but ranked second in citations (TC = 4,594). The United States leads in both total citations (TC = 7,928) and H-index (n=48). Harvard University ranks first among institutions in publications(n=34). Australia, ranked third, published only 50 articles on WMH but achieved the highest citation per article (CPA = 49.32).

**Table 3:** Top 10 countries and institutions in the field of WMH.

Country/Region	Articles (%)	TC	H-Index	CPA	Top institutions*	Top institution articles
China	202 (37.687%)	4,594	38	22.74	Chinese University of Hong Kong	22
USA	174 (32.463%)	7,928	48	45.56	Harvard University	34
Australia	50 (9.328%)	2,466	24	49.32	Griffith University	9
England	42 (7.836%)	1,872	20	44.57	University of London	13
Canada	38 (7.090%)	1,377	17	36.24	University of Toronto	8
South Korea	16 (2.985%)	532	10	33.25	Kyung Hee University	3
Spain	15 (2.799%)	363	9	24.2	University of Granada	3
Italy	14 (2.612%)	244	6	17.43	Catholic University of the Sacred Heart Marche Polytechnic University Sapienza University Rome University of Padua	2
Germany	11 (2.052%)	353	6	32.09	Charité Universitätsmedizin Berlin Free University of Berlin Humboldt University of Berlin Technische Universität Dresden University of Hamburg	2
Taiwan	11 (2.052%)	215	8	19.55	Chang Gung University	3

TC, Total Citation; CPA, Citations Per Article; *, Parallel institutions publish the same number.

### 3.4 Scholars and collaborative networks

**Figure 5:**
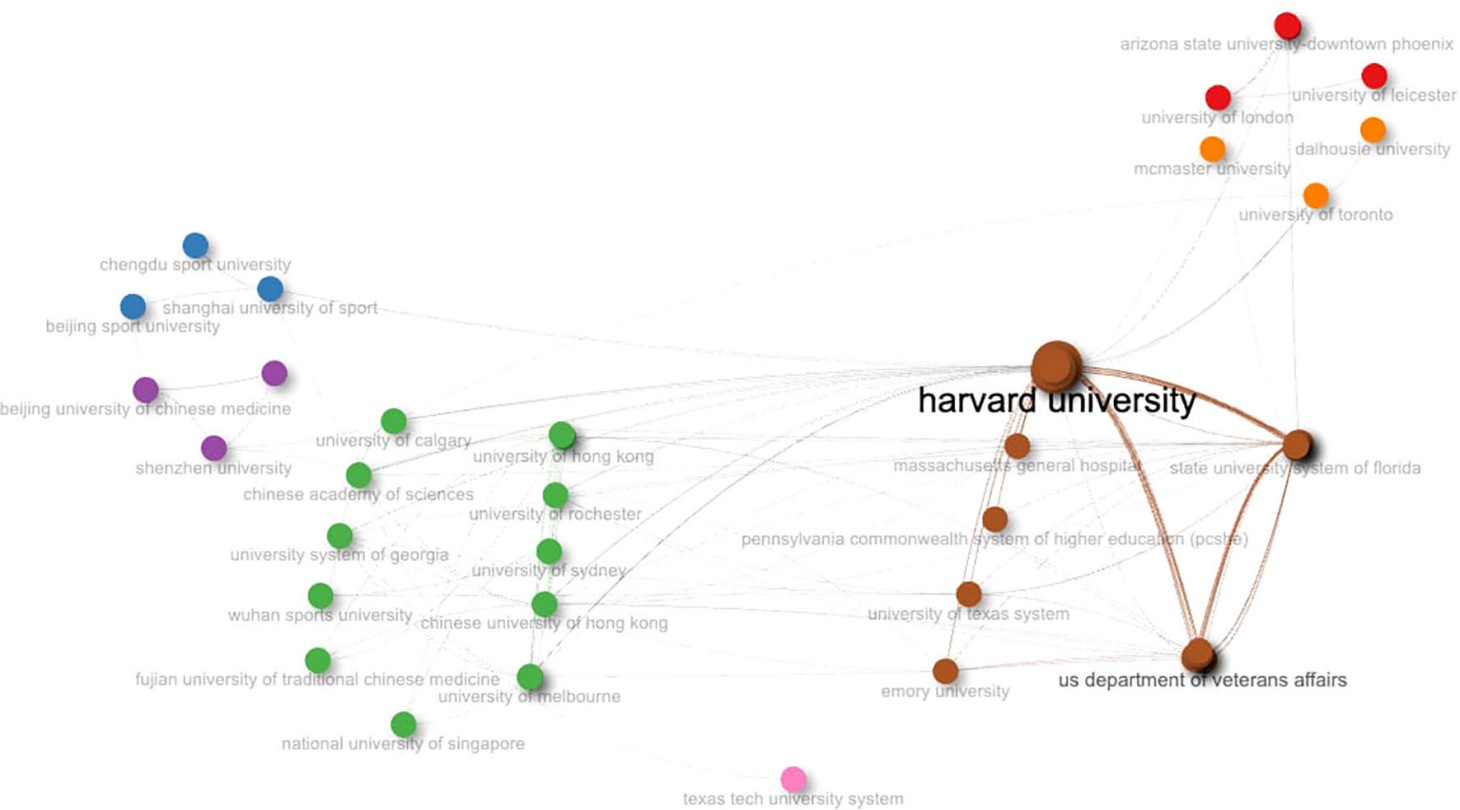
Global institutional partnerships in the WMH field.

**Figure 5** presents a network of institutional collaborations, revealing clusters of relationshipsamong different research teams. Through active collaboration with numerous international institutions, Harvard University has maintained a central position in its cooperative relationships. This role is quantitatively supported by its highest normalized cumulative degree and betweenness centrality in the network (**Supplementary Table S1**). This illustrates that the institution maintains extensive direct collaborative relationships and performs a crucial bridging function among different institutional clusters. Additionally, the blue cluster in the figure indicates close cooperation among Chinese institutions specializing in physical education, including Beijing Sport University, Shanghai University of Sport, and Chengdu Sport University. This model may be driven by resource concentration and international project leadership. It reflects a trend in global scientific collaboration where knowledge and academic influence cluster around central nodes.

In fact, the authors’ productivity analysis identified the top 10 contributors in the WMH field (**Table 4**). Wayne, Peter M. (USA) ranked first with 12 publications, followed by Wang, Chenchen (China, 11 publications) and Yeh, Gloria (USA, 9 publications). Regarding citation impact, Wang, Chenchen holds the highest total citations (TC = 560) with the H-index of 7. However, Chan, Aileen Wai Kiu (China) achieved the highest Citations Per Article. (n=54.29). Overall, four of the top 10 authors are affiliated with U.S. institutions, while six are from Chinese research organizations.

**Table 4:** Top 10 authors in the field of WMH.

Author	Country	Articles	H-index	TC	CPA
Wayne, Peter M.	USA	12	8	192	16
Wang, Chenchen	China	11	7	560	50.91
Yeh, Gloria	USA	9	6	351	39
Chan, Aileen Wai Kiu	China	7	7	380	54.29
Taylor-Piliae, Ruth E.	USA	7	7	373	53.29
Wang, Y	USA	7	6	207	29.57
Guo, Yan	China	6	5	130	21.67
Wei, Gao Xia	China	6	5	252	42
Zhang, Yue	China	6	4	122	20.33
Chen, Li Dian	China	5	5	116	23.2

TC, Total Citation; CPA, Citations Per Article.

**Figure 6:**
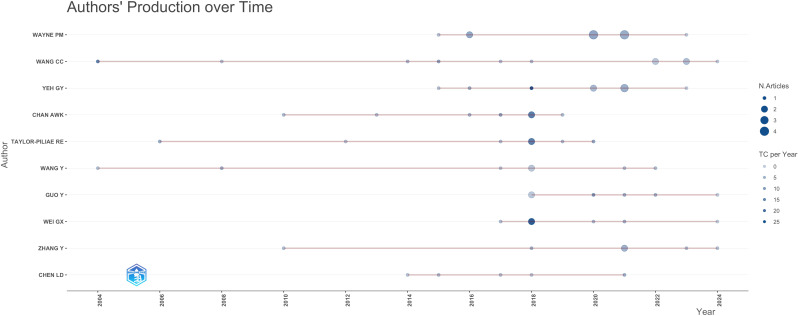
Publication timeline of the top 10 authors in the WMH field.

**Figure 6** shows the timeline of productivity for the 10 authors. Circle size represents the number of articles, while color intensity indicates annual citation counts. Wang, Chenchen has engaged in WMH research for over 20 years, followed by Wang, Y. from the United States in second place. In contrast, Wayne, Peter M. achieved the highest H-index (n=8) despite exploring WMH topics for only 10 years. Half of these top 10 authors began focusing on this subject within the past decade.

### 3.5 Research hotspots and thematic evolution

**Figure 7:**
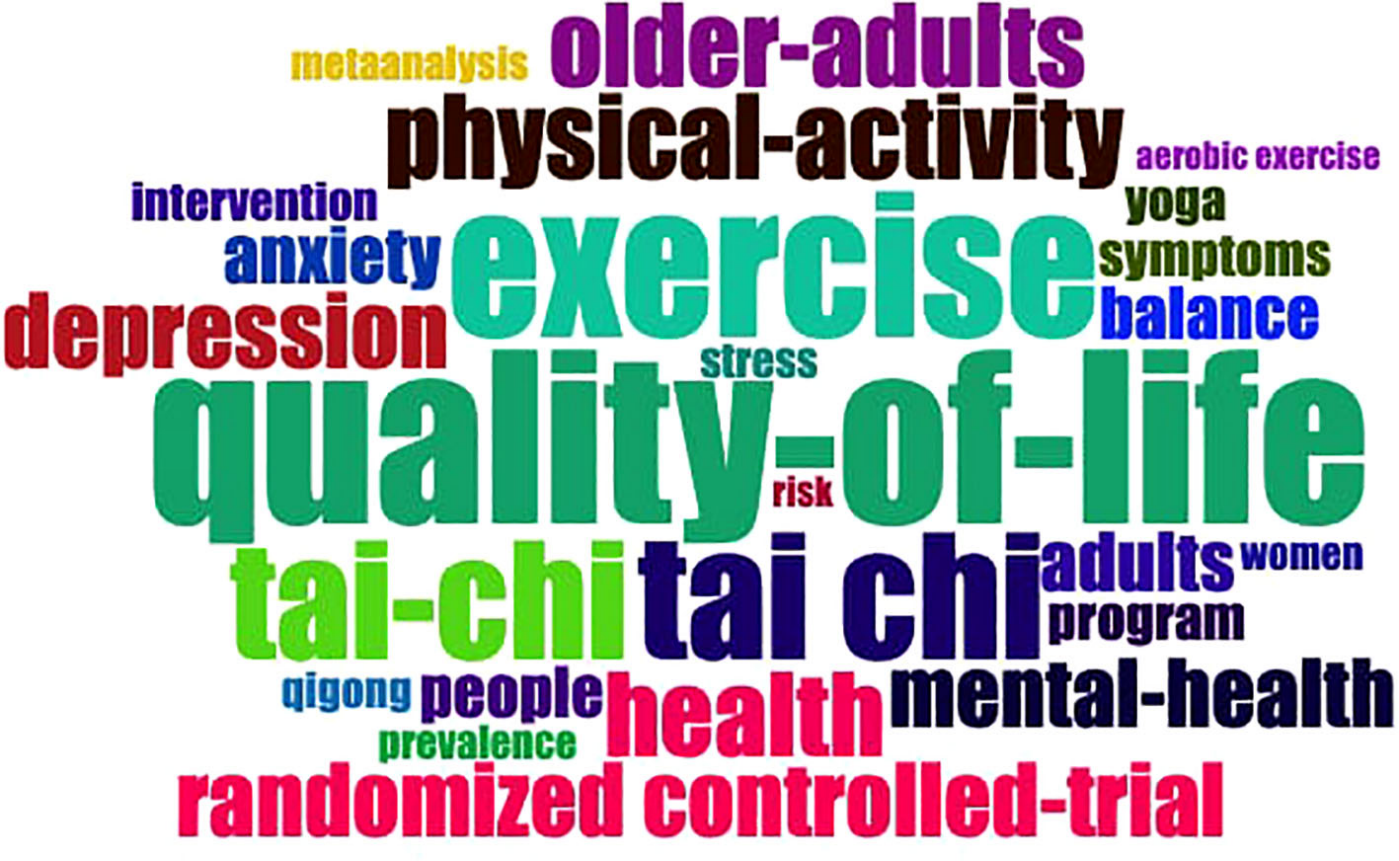
Word cloud map of WMH research.

Keyword analysis using Biblioshiny’s word cloud map provides an efficient overview of research hotspots in the WMH field. Compared to traditional frequency tables, word clouds can help readers quickly identify the most frequent topics (18). **Figure 7** shows the 25 most frequently occurring keywords in this field. The top keyword is quality-of-life (n=148), followed by exercise (n=124), tai chi (n=108), tai-chi (n=101), and physical-activity (n=73). Other frequently used terms include health (n=72), older-adults (n=72), depression (n=65), randomized controlled-trial (n=63), and mental-health (n=59).

**Figure 8:**
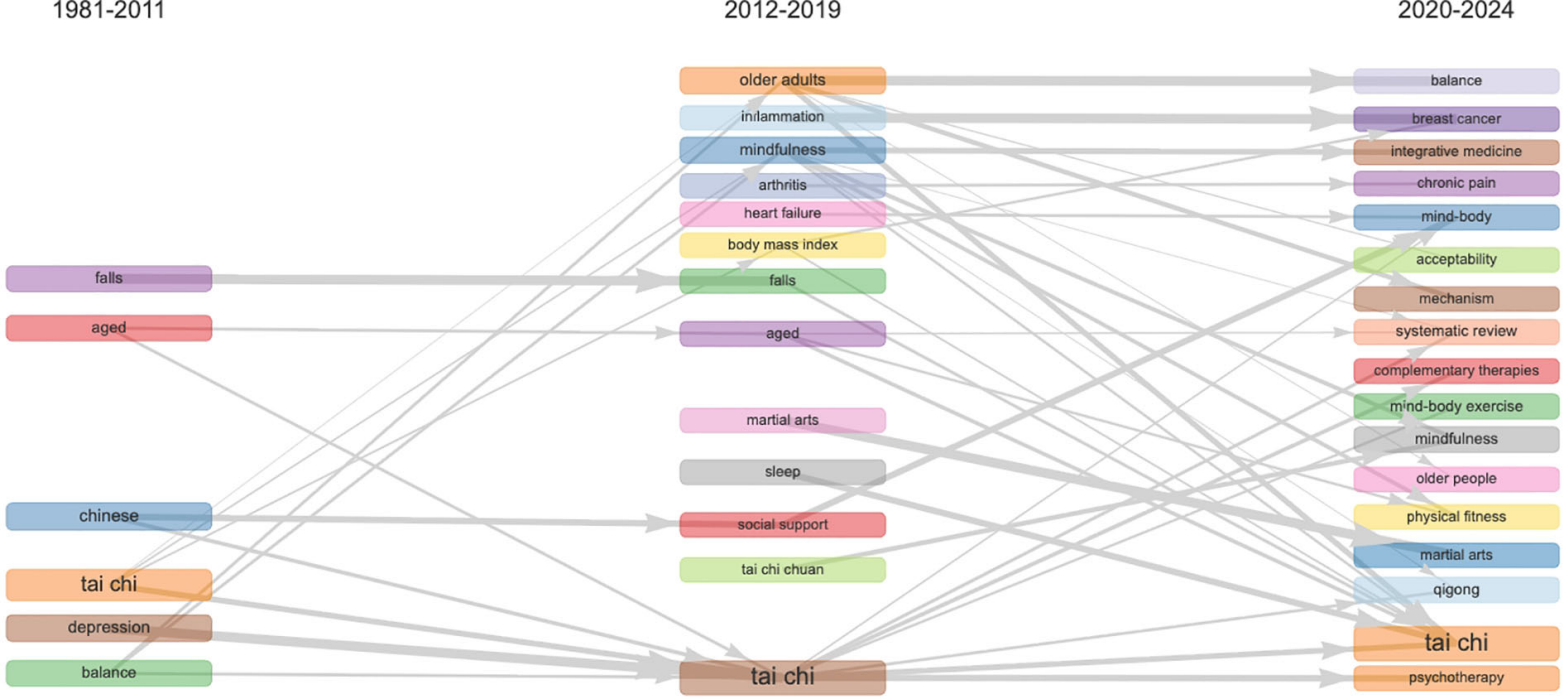
Thematic evolution of WMH research (1981–2024).

In bibliometric research, examining the evolution of keywords provides a critical perspective for identifying research trends, emerging themes, and the structural development of a field (17–19). By tracking the co-occurrence of terms across different periods, we can uncover the dynamic changes in academic focus (18–20). Biblioshiny offers a powerful analytical tool to visualize this process, generating intuitive thematic evolution diagrams that highlight the strength and stability of connections between keywords (17, 18). Using the previous two decades (2011) and the most recent five years (2019) as key date, we got a thematic evolution trend for the WMH field (**Figure 8**). Analysis of high-frequency keywords and thematic evolution across these three periods reveals the changing of WMH’s research interests.

#### 3.5.1 Aging and rehabilitation (1981–2011)

Early research primarily focused on terms such as “aged” (K. M. 31–33),”falls” (22, 33, 34), “balance” (22, 33, 34), and “depression” (32, 35). These keywords mainly addressed the aging population and the application of “Chinese Tai Chi” as a rehabilitation intervention (36, 37).

Meanwhile, the associations between terms are relatively weak. The fewer theme words present at this stage reflect that WMH research remains primarily in an exploratory phase, focusing on basic clinical performance and benefits rather than broader psychosocial outcomes. (36, 37).

This early thematic cluster shares certain intrinsic connections with contemporary rehabilitation research. Researchers were more focused on their functional outcomes. Tai Chi and related Wushu exercises were introduced into Western health studies primarily as low-risk physical interventions. They were employed to prevent falls among the elderly and maintain functional abilities, rather than mainly as practices for mind-body integration or mental health.

#### 3.5.2 Mental health and mind–body (2012–2019)

This period expanded the scope of health topics while continuing previous themes such as “aged” (38, 39),”falls” (38, 40), and “tai chi” (39, 41). New additions included: “arthritis” (41, 42), “body mass index” (43, 44), “heart failure” (40, 43), “inflammation” (41, 42),and “sleep” (45). Naturally, the exercise patterns were refined to include “martial arts” (40), “mindfulness” (46), and “tai chi chuan” (47).

Meanwhile, this period strengthened interconnections between keywords, revealing the diversity of research topics (40, 47). This thematic expansion reflected the broader shift within health research toward a mind-body integration framework. Researchers began recognizing the interactions among physiological conditions, mental health, and lifestyle factors. With the growing influence of integrative medicine and mindfulness interventions, WMH made a unique contribution to the diversification of global mental health research themes.

#### 3.5.3 Integration and public health (2020–2024)

The latest phase established a direct link between “tai chi” and “psychotherapy” (48, 49). The emergence of keywords like “breast cancer” (50, 51) and “chronic pain” (52, 53)indicates new connections between WMH and chronic disease management (11).

Furthermore, the appearance of terms such as “acceptability” (54), “complementary therapies” (14, 55)”integrative medicine” (56, 57) and “mechanism” (3, 58) deepens WMH’s status within public health research. The emergence of “systematic review” (48, 49, 56) indicates that the field of WMH research has produced a growing number of evidence-based research articles and increasingly emphasizes the synthesis of evidence (59).

This shift aligns with macro trends in global health research, including increased emphasis on evidence-based practice, mental health promotion, and non-pharmacological interventions. When external pressures such as aging societies and the COVID-19 pandemic emerged, they accelerated the integration of mental health research into public health frameworks. Within this trend, more researchers began focusing on the systematic integration of action mechanisms, acceptability, and clinical data.

### 3.6 Clinical progress analysis (2020–2024)

The PubMed database focuses on clinical research and directly demonstrates the practical effects of Wushu interventions (such as Tai Chi and Qigong) in the field of mental health. The inclusion criteria were clinical trial publications from 2020 to 2024. Finally, this study included a total of 23 relevant WMH studies.

#### 3.6.1 General characteristics of research

**Table 5** shows the general characteristics of clinical research in WMH field over the past five years. Interestingly China and the United States remain the leading countries in clinical research, contributing 11 items (51, 60–69) and 7 items (70–76) respectively, accounting for nearly 80% of the total. The randomized controlled trials (RCTs) were the most popular research design, accounting for 78.2% (n=18) (51, 60–62, 64–67, 69–73, 75, 77–80). However, study sizes were generally limited, with nearly 70% of studies having sample sizes below 100 participants (51, 60, 62, 64–67, 69–71, 74, 75, 77, 79–81), and nearly half of these studies having sample sizes under 50 participants (51, 62, 67, 69, 74, 77, 79–81).

**Table 5:** General characteristics of research.

Characteristic category	Specific classification	Number of studies (%)
Research Country	China	11 (47.8%)
	The United States	7 (30.4%)
	The United Kingdom	2 (8.6%)
	Australia	1 (4.3%)
	South Korea	1 (4.3%)
	Iran	1 (4.3%)
Research Design	Randomized Controlled Trial (RCT)	18 (78.2%)
	Pilot/Feasibility study	3 (13.0%)
	Prospective cohort	1 (4.3%)
	Cross-sectional study	1 (4.3%)
Sample Size	n < 50	9 (39.1%)
	50 ≤ n < 100	7 (30.4%)
	n ≥ 100	5 (21.7%)
Intervention Type	Tai Chi	20 (86.9%)
	Qigong or Mixed mind-body training	3 (13.0%)
Intervention Duration	n < 8 weeks	2 (8.6%)
	8 ≤ n ≤ 12 weeks	13 (56.5%)
	n >12 weeks	6 (26.0%)
Intervention Frequency	Per day	2 (8.6%)
	Once a week	1 (4.3%)
	Twice a week	6 (26.0%)
	Three times a week	7 (30.4%)
	Four to five times a week	4 (17.3%)
	Irregular	4 (17.3%)
Intervention Format	Face-to-face instruction	15 (65.2%)
	Remote online instruction	2 (8.6%)
	Mixed instruction	3 (13.0%)

In the recent intervention program, Tai Chi is the dominant intervention form, and 86.9% of the studies have adopted it (60–66, 68–80). Meanwhile, a moderate intervention period of 8–12 weeks is the general choice of researchers (n=13) (51, 61, 62, 65, 66, 69, 71–73, 77–80), and usually follows the training frequency of 2–3 times a week (51, 61, 62, 66, 69–73, 77–80). Moreover, the traditional face-to-face instruction remained the major trend approach, accounting for 65.2% (n=15) (51, 61, 62, 65, 66, 68, 69, 71, 72, 75, 77–81). However, remote online instruction (n=2) (64, 73) and mixed learning models have begun to emerge (n=3) (60, 67, 70).

#### 3.6.2 Clinical research focus and main achievements

As shown in **Table 6**, through systematic analysis of the 23 included clinical studies, this research identified five primary areas of clinical application in the WMH research field: mental health and emotional adjustment (51, 61, 62, 65–69, 74, 77, 78, 81), chronic pain and rehabilitation (70–72, 76), neurological and cognitive health (65–67, 81), cancer and chronic disease support (51, 60–62, 69, 71–73, 75), and public health promotion (63–65, 69, 73, 75, 77, 78). Among these, mental health and emotional regulation (n=12) received the highest number of studies, primarily focusing on improving anxiety, depression, stress, and well-being. Secondly, researchers pay attention to related research in rehabilitation of chronic diseases (n=9) and public health (n=8).

**Table 6:** Clinical research focus and main achievements.

Key clinical areas	Representative conditions	Number of studies	Primary evaluation indexes	Main research results
Mental health and emotional adjustment	Anxiety, Depression, Stress, Well-being	12	HADS, CES-D, GAD-7, SWB, PSQI	Tai Chi or mind-body interventions significantly improved anxiety, depression, and sleep quality
Chronic pain and rehabilitation	Chronic Musculoskeletal Pain, COPD, Arthritis	4	CRQ, PGIC, SF-36	Safe and feasible; significantly enhanced emotional functioning and quality of life
Neurological and cognitive health	Attention, Theta Brain Waves, Executive Function	4	EEG, EF Task, EMA, FESI	Tai Chi significantly increased prefrontal Theta activity and executive control
Cancer and chronic disease support	Breast Cancer, Lung Cancer, PICC Cancer	9	HADS, PSQI, QoL, BFI	Significant improvement in anxiety, fatigue, and quality of life
Public health promotion	Hypertension, Isolated Populations	8	CD-RISC, SF-20-MH, WEMWBS	Enhanced psychological resilience, well-being, and social connectedness

HADS, Hospital Anxiety and Depression Scale; CES-D, Center for Epidemiological Studies Depression Scale; GAD-7, Generalized Anxiety Disorder Scale; SWB, Subjective Well-Being Scale; PSQI, Pittsburgh Sleep Quality Index; COPD, Chronic Obstructive Pulmonary Disease indicator; CRQ, Chronic Respiratory Questionnaire; PGIC, Patient Global Impression of Change; SF-36, Short Form Health Survey; EEG, Electroencephalography; EF Task, Executive Function Task; EMA, Ecological Momentary Assessment; FESI, Falls Efficacy Scale International; PICC, Peripherally Inserted Central Catheter–related assessment; QoL, Quality of Life; BFI, Brief Fatigue Inventory; CD-RISC, Connor-Davidson Resilience Scale; SF-20-MH, Short Form Health Survey Mental Health Subscale; WEMWBS, Warwick-Edinburgh Mental Well-being Scale.

Their research results show that Chinese Wushu (Tai Chi, Qigong and other training methods) can effectively improve mental health across different populations. Benefits include reduced anxiety and depression levels, enhanced well-being, relieved fatigue, and improved sleep quality (51, 60–62, 65, 66, 68, 69, 71, 74, 75, 77, 78, 81). Meanwhile, some studies also combined physiological and biochemical indexes (e.g. HRV, Hormone, and EEG) (66, 74, 80) to verify the effect of Wushu intervention on autonomic nerve regulation. However, the mental health measurement tools employed in these studies vary widely, lacking a unified evaluation framework.

## 4 Discussion

### 4.1 Growing interest for WMH

This bibliometric analysis indicates a significant increase in research on WMH, particularly over the past decade. The stable growth in publications and citations shows that WMH has become an emerging interdisciplinary field attracting global attention (48, 49). The primary driver of this trend is the growing recognition that mind-body interventions such as Tai Chi and Qigong offer effective non-pharmacological strategies for improving mental health outcomes (3, 52, 57, 59, 82). For instance, the WHO *Global Action Plan on Physical Activity 2018–2030* highlights traditional exercises, including Tai Chi, as promising approaches for promoting mental health (83). The peak of publications in recent years is related to the high interest in non-pharmaceutical interventions during the COVID-19 epidemic, when keeping social distance and mental health crisis stimulated people’s demand for accessibility (84). To sum up, these findings demonstrate that WMH research has gained great academic development motivation, reflecting its growing importance in the scientific communities.

### 4.2 Core journals and articles

A series of core journals and highly cited publications were identified through bibliometric analysis, forming the knowledge foundation of WMH research. As shown in **Figure 3**, this concentration emphasizes the crucial position of a small number of journals in spreading WMH academic achievements. The top ten journals span a broad disciplinary range, including psychology (multidisciplinary) (4, 7, 8), medicine (general practice and internal medicine) (52, 58), integrative (13, 55) and complementary medicine (3, 14, 39, 85, 86), public environmental and occupational health (56, 87), multidisciplinary sciences, and psychiatry (88).

This distribution indicates that WMH research is not a single discipline, but rather an interdisciplinary science spanning psychology, medicine, and public health. Some journals in **Table 1** belong to fully open-access publishers, such as Frontiers Media SA (7), MDPI (84), and Hindawi LTD (85). Among the top ten most influential journals, three belong to Frontiers Media SA. This open-access publishing model increases the visibility of WMH research and facilitates the rapid dissemination of its findings.

Furthermore, analysis of the most influential publications indicates that systematic review methodologies account for a significant proportion of WMH research, reflecting the importance of synthesizing research evidence (12, 23–25, 27). The *Cochrane Database of Systematic Reviews* holds significant influence among journals publishing reviews, and its research findings are frequently accepted by scholars. (23, 89). The most highly cited article in **Table 2**, published in 1996, explored that Tai Chi effectively reduces fear of falling among older adults (22). Through randomized controlled trials, it has confirmed the positive effects of Wushu training in psychological interventions (22). It has provided the experimental evidence foundation for WMH research and indicated the practical legitimacy of Wushu in clinical trials.

### 4.3 Global contribution pattern

It is evident that half of the ten most cited publications in WMH research originate from U.S. institutions, reflecting America’s leading position in this field (22, 25–28). Simultaneously, the geographical distribution of publications (**Figure 4**) further confirms the prominent contributions from the United States. However, **Table 3** reveals disparities in productivity and impact among different countries. China accounts for the largest share of total publications (37.7%) but its citation rate (CPA = 22.74) follows the United States. Despite ranking second in publication volume, the United States demonstrates significantly enhanced academic influence (CPA = 45.56, H-index=48). This contrast indicates that China focuses on expanding the volume of WMH research, while the United States maintains its influence through widely cited systematic reviews and clinical trials.

It is noteworthy that countries such as Australia and the United Kingdom, despite producing fewer publications, exhibit unusually high citations per article (CPA>44). This reflects the significant impact of high-quality research from institutions like Griffith University (43, 48) and the University of London (88, 89). Additionally, Germany demonstrates the role of leading medical centers (Charité Universitätsmedizin Berlin) in enhancing national popularity(Esch 90). The integrated model combining universities and medical centers actively promotes WMH research progress. However, WMH research is still geographically unbalanced, development concentrated in a few academic centers, and globally large regions lack adequate representation in this field.

### 4.4 Collaboration networks and key author

The institutional collaboration network (**Figure 5**) highlights the appearance of a unique knowledge cluster in WMH research. Harvard University stands as a central hub connecting numerous institutions across North America, Europe, and Asia (28). This network of links reflects the interdisciplinary and transnational nature of WMH research, bringing together medical schools, public health institutions, and traditional Chinese medicine universities within this field (28). This collaborative model covers a broader range of participants, integrate different research methodologies, and expands cross-cultural perspectives on Wushu interventions (28). However, the network remains fragmented, with most institutions concentrated within national or regional boundaries.

Regarding author productivity (**Table 4**, **Figure 6**), top contributors exhibit geographic and time concentration. Wang, Y. (25) and Ruth E. Taylor-Piliae (91) were among the earliest scholars in the United States to publish WMH articles and establishing stable citation impact. In contrast, Wang Chenchen from China is the most highly cited researcher in this field (TC = 560) and has demonstrated active publishing in recent years (92). Although China and the United States have profound academic influence in this field, dependence on a fewer number of highly productive authors will limit the diversity of perspectives. Future research should promote broader participation from underrepresented regions and interdisciplinary scholars, thereby building a more balanced research community.

### 4.5 Thematic evolution and emerging research

To assist researchers in systematically understanding the thematic evolution within the field of WMH, this study outlines the knowledge development landscape by analyzing its intrinsic logic. Based on keyword co-occurrence analysis, we organize the knowledge structure of WMH from a psychological perspective, thereby illuminating the progression of core themes. The following discussion will explore these insights across three psychological dimensions, while also exploring research gaps and future directions.

#### 4.5.1 Three psychological dimensions

As shown in **Figure 7**, the word cloud reflects the multidimensional integration characteristics of WMH research: using Tai Chi as the primary intervention form to improve quality of life and mental health through mind-body regulation mechanisms. Furthermore, the distribution of keywords indicates that WMH research mostly focuses on three thematic domains. First, Wushu-related exercise modalities such as tai chi (108), tai-chi (101), and qigong (31) constitute foundational interventions (4, 93). Second, mental health outcomes form a core research category, including quality-of-life (148), depression (65), mental-health (59), anxiety (49), and stress (31), highlighting these practices’ central objectives (13, 24, 44, 49, 90). The third theme is methodological approaches, specifically randomized controlled trials (63) and meta-analyses (29), emphasizing the importance of evidence-based validation (38, 94). These keywords reveal how WMH research integrates traditional practices with modern scientific to address mental health challenges.

The longitudinal evolution of keywords (**Figure 8**) reveals the thematic and time changing in WMH research. The field’s thematic evolution can be understood through three primary psychological dimensions:

##### 4.5.1.1 Chronic disease management and aging dimension

Covering the older adult (35, 39), chronic pain (26, 53), and falls (22, 33, 34). It showed that studies tended to explore how Wushu indirectly reduces mental burden by improving physical functions such as balance (22, 29, 89). Clinical trials confirm this theoretical hypothesis. For example, studies on COPD patients confirmed that Tai Chi effectively improved depressive symptoms (71). Similarly, research with chronic pain veterans and heart rehabilitation patients confirms that consistent Tai Chi practice enhances emotional regulation and self-efficacy (70, 76). Cross-sectional findings confirmed Wushu’s value in chronic disease management beyond physical rehabilitation, improving patients’ mental health outcomes.

##### 4.5.1.2 Mental disorders dimension

Including depression (13, 24), anxiety (24, 49), and stress (90, 95), these represent the most common psychological outcomes. It reflected researchers’ interest in studying Wushu as a non-pharmacological treatment for mental health (24, 32, 35). Results from most randomized controlled trials show that exercise interventions involving Tai Chi or Qigong effectively reduce participants’ anxiety, depression, and stress levels (51, 61, 62, 65–69, 74, 77, 78, 81). Furthermore, Wushu interventions have been found to be positively effective in various populations, including healthy college students (66), elderly people suffering from depression (68), and cancer patients (51, 61). These trial conclusions confirm the cross-group universality of WMH, providing clinical evidence for its use as a broad-spectrum mental health intervention.

##### 4.5.1.3 Well-being and social connection dimension

Involving quality of life (27, 44), mindfulness (51, 95), and social support (54). It represents a shift in the WMH field from traditional disease treatment models to positive health promotion approaches (28, 31). Empirical studies on well-being indicate that Wushu practices enhance quality of life and mental wellbeing among community elders and hypertensive patients (74). Furthermore, the communal context created by group Wushu exercises can promote social connection and help participants fight isolation (65, 70). These clinical findings show Wushu’s unique value in promoting public health and building social networks.

From a timeline perspective, aging and rehabilitation (1981–2011) marked the initial phase of WMH research. Studies primarily focused on issues such as aged, falls, and balance. It reflects that Wushu related exercises (especially Tai Chi) were originally applied to the elderly as a low-impact rehabilitation strategy (22, 33, 34). The high co-occurrence among wushu, aging and fall shows that mental health findings were not originally investigated as isolated psychological structures (31–33). On the contrary, they are embedded in functional and rehabilitative indicators (33, 34), such as balanced confidence (22), fear of falling (22, 33) and overall happiness (31). This structural embedment explains why the subject of gerontology occupies the core position in the thematic evolution.

Mental health and mind–body (2012–2019) represented diverse characteristics. Research scope has expanded to psychological level outcomes, incorporating concepts such as mindfulness, sleep, and social support, reflecting the integration of Wushu with broader mind-body therapies (45, 46). Finally, integration and public health (2020–2024) marks a change toward a comprehensive approach. Researchers consider Wushu as a mind-body exercise (88), adding it into integrative medicine or complementary therapies to address public health challenges like chronic pain and cancer (51–53, 96). This thematic evolution reflects the transformation of WMH’s research interests and its progression in the field of global public health applications.

Moreover, this study compared the results of bibliometrics with clinical trials reports. Over the past five years, the research topics of clinical trials have shown high consistency with bibliometric hotspots. The most frequently appearing clinical issues include quality of life, anxiety, depression, sleep quality, and health promotion in the older adult population (51, 61, 62, 65–69, 74, 77, 78, 81). Meanwhile, Tai Chi has been the most used intervention form (60–66, 68–80).

In summary, **Figures 7**, **8** collectively illustrate how WMH research has evolved from an early focus on physical rehabilitation to a multidisciplinary field centered on mental health promotion. This progression reveals a psychological orientation within Wushu studies, providing a theoretical foundation for understanding its multidimensional mechanisms of emotional adjustment, cognitive improvement, and social support.

### 4.6 Research gaps and future directions

Despite significant progress in WMH research over the past four decades, this rapid development has simultaneously exposed several critical limitations. Current research remains limited in theoretical depth, diversity of interventions, cultural adaptability, and international collaboration. They constrain theoretical development in the field but also provide new directions for future research to explore.

#### 4.6.1 Mechanistic limitations

Existing WMH research mostly relies on self-report instruments or behavioral performance to evaluate intervention efficacy (35, 44), lacking in-depth exploration of the underlying neurophysiological mechanisms (97). While this research approach validates the effectiveness of Wushu, it fails to fully explain the reasons behind this efficacy. The unique breathing regulation, attention focus, and body awareness training in Wushu may influence emotional stability by regulating the autonomic nervous system, the prefrontal-limbic pathway, and the interoceptive network (37). Future research should integrate multimodal approaches such as neuroimaging, psychophysiological indicators, and laboratory cognitive tasks. This will reveal action pathways from perspectives including neuroplasticity, emotion regulation mechanisms, and mind-body integration mechanisms. This mechanistic research will enrich theoretical models for mental health interventions and promote the status of Wushu within health science systems.

#### 4.6.2 Imbalance of intervention forms

Tai Chi has almost monopolized the intervention forms in existing WMH research, while other representative Chinese traditional Wushu such as Qi Gong, Xingyi Quan, Bagua Zhang, and Zhan Zhuang have rarely been systematically explored (3, 31). This singular focus weakens the understanding of the psychological value of the entire Wushu system and limits the scientific potential for cross-form comparisons (93). Future research should breach this Tai Chi centric framework. While preserving the core traditional philosophy, it should explore the differential effects of various internal and external Wushu styles, movement structures, and meditation intensities on psychological outcomes. Comparative research designs will reveal the common mechanisms or unique advantages of Wushu interventions. This approach will promote scientific validation of Wushu diversity and provide a foundation for constructing a spectrum of Wushu psychological interventions.

#### 4.6.3 Insufficient cross-cultural research

Existing WMH research has been primarily led by Chinese and American institutions, with study samples concentrated on East Asian or North American populations (28, 96). Cross-cultural comparisons and multicultural adaptability studies remain relatively scarce. As a cultural practice combining physical training and philosophical cultivation, the psychological effects of Wushu may differ across cultural contexts (9, 31). Future research should strengthen transnational collaborations across cultural backgrounds to compare how differences in motivation, beliefs, and bodily experiences between Eastern and Western individuals influence intervention outcomes. Meanwhile, creating cross-cultural psychological assessment instruments and translated adaptive scales will enhance the external validity and global applicability of research. By advancing such comparative cultural studies, Wushu may evolve from a localized tradition into a globally shared mental health intervention model.

#### 4.6.4 Methodological and collaborative limitations

Although the increase in RCTs and meta-analyses indicates that WMH research is maturing methodologically, existing studies are generally small in scale (85), insufficiently heterogeneous in samples (86, 94), short in follow-up duration (7), and inconsistent in outcome measurement standards (48). These factors limit the reliability and replicability of conclusions. Smaller sample sizes may reduce statistical power. Heterogeneity in mental health assessment tools will limit comparability across studies. These methodological limitations illustrate the need for more rigorous and standardized clinical trials in future research.

Furthermore, research collaboration still exhibits regional clustering, with loose international networks and a lack of shared databases. Future efforts should focus on establishing multi-institutional, transnational collaborative research platforms, setting standardized research protocols, and implementing shared data repositories with open-access mechanisms to enhance research reproducibility.

Overall, the thematic evolution of WMH research shows a profound transformation from rehabilitation orientation to comprehensive mental health and public health perspectives. Future studies should progress from examining effectiveness to exploring the mechanisms of why interventions are effective, from single interventions to multiple comparative approaches, from localized experiments to cross-cultural integration, and from independent research to global collaboration. This is the ideal way to truly enhance the scientific, widespread, and sustainable development of Chinese Wushu in the international mental health care field.

## 5 Conclusion

Through a comprehensive analysis of global research findings from 1981 to 2024, this study reveals the overall development trends, core contributors, research hotspots, and knowledge structure within the WMH field. Results indicate a significant growth in WMH research following COVID-19. The most influential countries, institutions, journals, articles, and authors are the United States, Harvard University, Frontiers in Psychology, *Reducing frailty and falls in older persons: An investigation of Tai Chi and computerized balance training*, and Wayne, Peter M. Other Chinese Wushu interventions similar to Tai Chi will likely emerge as future trends.

However, international collaboration networks still exhibit characteristics of national isolation, with limiting knowledge sharing. The reliance on highly cited systematic reviews reveals a shortage of systematic and longitudinal research in this field, which needs breakthroughs in standardizing intervention models, enhancing cross-cultural adaptability, and promoting interdisciplinary integration. In summary, this study constructs a knowledge diagram and evolutionary pathway for the WMH field. It provides researchers with a reference for clarifying the research foundation, identifying key gaps, and recognizing collaborative networks. It offers scientific evidence for organizations and policymakers to support the sustainable development of Chinese Wushu in global mental health promotion.

## 6 Limitation

This study has certain limitations. Bibliometric analysis, while useful for mapping broad patterns, cannot capture the depth or quality of individual studies, which future researchers may address through qualitative reviews. In addition, limiting the analysis to English-language publications may introduce language bias, particularly considering extensive Chinese-language research on wushu-related practices. This points to an urgent need to include multilingual and cross-cultural databases. Finally, dependence on a single database may result in insufficient coverage. While WoSCC is a widely used and authoritative database, reliance on it alone risks excluding relevant publications indexed elsewhere, such as in Scopus. Future studies are encouraged to integrate multiple sources such as Scopus, PubMed, and regional repositories to provide a more comprehensive overview.

## Data Availability

Publicly available datasets were analyzed in this study. This data can be found here: https://pubmed.ncbi.nlm.nih.gov/?term=%28%22Wushu%22%5Btiab%5D+OR+%22Chinese+Martial+Arts%22%5Btiab%5D+OR+%22Kung+Fu%22%5Btiab%5D+OR+%22Gongfu%22%5Btiab%5D+OR+%22Taijiquan%22%5Btiab%5D+OR+%22Tai+Chi%22%5Btiab%5D+OR+%22Tai+Chi+Chuan%22%5Btiab%5D+OR+%22Sanda%22%5Btiab%5D+OR+%22Chinese+Kickboxing%22%5Btiab%5D%29+AND+%28%22Mental+Health%22%5Btiab%5D+OR+%22Mental+Illness%22%5Btiab%5D+OR+%22Psychology%22%5Btiab%5D+OR+%22Psychosocial%22%5Btiab%5D+OR+%22Wellbeing%22%5Btiab%5D%29+AND+%22humans%22%5BMeSH%5D&filter=pubt.clinicaltrial&filter=years.2020-2024 https://www.webofscience.com/wos/woscc/summary/78297be4-fc14-4abc-95a6-5b344b09e743-0186bbec00/relevance/1.

## Supplementary material

Supplementary Table 1 Centrality metrics of the top 10 institutions in the WMH collaboration network. Cumulative Degree represents the normalized degree centrality of each institution in the collaboration network, reflecting the relative number of direct collaborative links compared with the most connected institution (scaled to a maximum value of 1). Betweenness indicates the extent to which an institution lies on the shortest collaboration paths between other institutions, capturing its role as a bridge or intermediary within the global WMH research network.
