# Supplementary material for: A bibliometric and visualization analysis for global research trends in Wushu and mental health (1981–2024)
Source: Front Psychiatry. 2026 Feb 13;17:1737574. doi: 10.3389/fpsyt.2026.1737574 (PMC12946104; doi:10.3389/fpsyt.2026.1737574)
Supplement: Supplementary Table 1 — Centrality metrics of the top 10 institutions in the WMH collaboration network. Cumulative Degree represents the normalized degree centrality of each institution in the collaboration network, reflecting the relative number of direct collaborative links compared with the most connected institution (scaled to a maximum value of 1). Betweenness indicates the extent to which an institution lies on the shortest collaboration paths between other institutions, capturing its role as a bridge or intermediary within the global WMH research network. [file Table1.docx]

| Supplementary Table S1 Centrality metrics of the top 10 institutions in the WMH collaboration network. | | |
| --- | --- | --- |
| Institution | Betweenness | Cumulative Degree |
| Harvard University | 310.321 | 1 |
| Harvard Medical School | 125.055 | 0.809 |
| University of Toronto | 91.374 | 0.111 |
| Harvard University Medical Affiliates | 87.225 | 0.876 |
| Shanghai University of Sport | 86.94 | 0.138 |
| Shenzhen University | 81.06 | 0.107 |
| Chinese University of Hong Kong | 73.573 | 0.311 |
| University of Calgary | 70.453 | 0.098 |
| University of California System | 65.823 | 0.333 |
| University System of Georgia | 57.224 | 0.111 |
| Notes: Cumulative Degree represents the normalized degree centrality of each institution in the collaboration network, reflecting the relative number of direct collaborative links compared with the most connected institution (scaled to a maximum value of 1). Betweenness indicates the extent to which an institution lies on the shortest collaboration paths between other institutions, capturing its role as a bridge or intermediary within the global WMH research network. | | |
